# Downregulation of Neuronal and Dendritic Connexin36-Made Electrical Synapses Without Glutamatergic Axon Terminals in Spinal Anterior Horn Cells From the Early Stage of Amyotrophic Lateral Sclerosis

**DOI:** 10.3389/fnins.2018.00894

**Published:** 2018-11-28

**Authors:** Yuko Kobayakawa, Katsuhisa Masaki, Ryo Yamasaki, Wataru Shiraishi, Shotaro Hayashida, Shintaro Hayashi, Koichi Okamoto, Takuya Matsushita, Jun-ichi Kira

**Affiliations:** ^1^Department of Neurology, Neurological Institute, Graduate School of Medical Sciences, Kyushu University, Fukuoka, Japan; ^2^Department of Neurology, Geriatrics Research Institute and Hospital, Gunma, Japan

**Keywords:** connexin36, amyotrophic lateral sclerosis, electrical synapse, chemical synapse, SOD1^G93A^ mouse

## Abstract

Connexin36 (Cx36) forms gap junctions between neurons, which are called electrical synapses, enabling adjacent neurons to communicate directly. The participation of chemical synapses in neurodegeneration in amyotrophic lateral sclerosis (ALS) has long been indicated, but it remains unclear whether electrical synapses are involved in the pathogenesis of ALS. We performed extensive immunopathological analyses using mutant superoxide dismutase 1 (SOD1^G93A^) transgenic mice and their littermates to investigate whether Cx36-made electrical synapses are affected in motor neuron diseases. We found that in the lamina IX of the lumbar spinal cord from wild type mice, about half of the Cx36 puncta existed independently of chemical synapse markers, while the rest coexisted with chemical synapse markers, such as vesicular glutamate transporter 1 (VGLUT1), which is a glutamatergic axon terminal marker, and/or glutamate decarboxylase 65 (GAD65), which is a GABAergic axon terminal marker. Cx36 single or Cx36/GAD65 double positive puncta, but not VGLUT1-containing puncta, were preferentially decreased on neuronal and dendritic surfaces of the anterior horn cells in the early stage of SOD1^G93A^ ALS mice. Moreover, in five human autopsied sporadic ALS cases with bulbar or upper limb onset, Cx36 immunoreactivity was diminished in the proximal dendrites and neuropils of well-preserved large motor neurons in the lumbar anterior horns. These findings suggest that downregulation of neuronal and dendritic Cx36 in the spinal anterior horns commonly occurs from the early stage of hereditary and sporadic ALS. Cx36-made electrical synapses without glutamatergic signaling appear to be more vulnerable than other chemical synapses and electrical synapses with glutamatergic signaling in the early stage of motor neuron degeneration, suggesting involvement of Cx36-made electrical synapses in the pathogenesis of human ALS.

## Introduction

Amyotrophic lateral sclerosis (ALS) is a neurodegenerative disease that affects the lower motor neurons in the brainstem and spinal cord as well as the upper motor neurons in the motor cortex. Loss of these neurons leads to muscle atrophy, weakness, fasciculations, and spasticity. Neuronal vulnerability in ALS has been investigated in considerable detail, and glutamate-induced excitotoxicity has been proposed to underlie ALS pathogenesis (Shaw and Ince, 1997; Heath and Shaw, 2002; Bosch et al., 2006). Excitotoxicity in neuronal synapses is induced by elevation in the extracellular glutamate concentration in the synaptic cleft or overstimulation of glutamate receptors on postsynaptic neurons (Bosch et al., 2006). In the central nervous system (CNS), there are two fundamentally different types of synapses: “chemical synapses,” including glutamatergic synapses, and “electrical synapses.” Although chemical synapses and electrical synapses closely interact and sometimes coexist as mixed synapses (Pereda, 2014), the involvement of electrical synapses in ALS pathogenesis is still unclear.

In the mammalian CNS, glial cells and neurons express cell-specific connexin (Cx) proteins (Rash et al., 2001a,b), which play important roles not only in maintaining homeostasis but also in pathological conditions (Dere and Zlomuzica, 2012; Belousov et al., 2017). Cxs are tetraspan integral membrane proteins, and their hexamers, called connexons, form head-to-head docking with other connexons of adjacent cells to construct gap junctions (GJs; Goodenough and Paul, 2009). In neuronal cells, adjacent neurons can directly communicate with each other by exchanging ions and small molecules through GJs, and this signal transduction mechanism is called an “electrical synapse” (Shimizu and Stopfer, 2013).

The primary Cx that constructs electrical synapses between neurons in the mammalian CNS is Cx36 (Condorelli et al., 1998; Connors and Long, 2004; Hormuzdi et al., 2004). Cx36 is expressed exclusively in neurons (Rash et al., 2001a,b) and is widely distributed in the mammalian CNS. In humans, *Cx36* mRNA is detected in various parts of the brain, such as the inferior olive, brainstem, hippocampus, cerebellar cortex, striatum, hypothalamus, and cerebral cortex (Belluardo et al., 1999; Condorelli et al., 2000; Long et al., 2000; Landisman et al., 2002; Placantonakis et al., 2006; Marshall et al., 2007; Cummings et al., 2008; Vervaeke et al., 2010). In the spinal cord, *Cx36* mRNA is detected in all the lamina of the gray matter and is predominant in the motor neurons of the anterior horn (Belluardo et al., 1999). The major function of the electrical synapse is neuronal synchronization that generates oscillatory activity; however, lateral spread and forward transmission also occur (Hormuzdi et al., 2001; Bennett and Zukin, 2004). During the acute phase of neuronal injuries in the rodent, such as ischemia, traumatic injury, epilepsy, and inflammation, the expression of Cx36 is transiently increased in units of a few hours (Oguro et al., 2001; Gajda et al., 2003; de Pina-Benabou et al., 2005; Garrett and Durham, 2008; Wang et al., 2012). Most of these studies support the death-promoting effect of Cx36-made GJs during glutamate-dependent neuronal death in the acute phase of neuronal injuries, but a few studies have indicated a prosurvival effect of Cx36 upregulation (Belousov and Fontes, 2013; Belousov et al., 2017).

We have previously reported that astrocytic and oligodendrocytic GJs in the anterior horn of the spinal cord in mutant superoxide dismutase 1 (SOD1^G93A^) transgenic mice were profoundly affected at the disease-progressive and end stages, where disruption of GJs among glial cells may exacerbate motor neuronal death (Cui et al., 2014). Recently, a decrease in Cx36 protein in whole spinal cord from SOD1^G93A^ ALS mice at the late stage [postnatal day 139 (P139)] and in autopsied human ALS cases was demonstrated by western blot analysis (Belousov et al., 2018). However, no morphological studies of Cx36 on neurons and dendrites have been reported. Thus, changes in Cx36-made GJs or electrical synapses in ALS remain to be established, especially in the early stages of the disease. Therefore, in the present study, we aimed to clarify fine morphological changes in Cx36-made electrical synapses and chemical synapses during the early stages of ALS, by investigating lumbar spinal cord anterior horn cells from SOD1^G93A^ transgenic ALS model mice as well as the morphologically well-preserved motor neurons in the lumbar spinal cord from bulbar or upper limb onset human autopsied ALS cases.

## Materials and Methods

### Animals

Transgenic mice with the human *SOD1^G93A^* gene [B6SJL-Tg(SOD1^∗^G93A)1Gur/J; Stock Number: 002726] (SOD1^G93A^ mice) were purchased from Jackson Laboratory (Bar Harbor, ME, United States). They were crossed with C57BL/6J mice over seven generations to maintain the strains, and hemizygous animals were examined in the experiments. These animals start to lose body weight around 12 weeks of age and exhibit apparent ALS-like symptoms, such as leg tremor, decreased stride, and muscle strength around 16 weeks of age. Death occurs at approximately 20 weeks (Yamasaki et al., 2010). We sampled transgenic mice at 8 weeks of age for the presymptomatic stage, 12 weeks of age for the onset stage, and 15 weeks of age for the progressive stage. Non-transgenic littermates were used as wild type mice. All animals were maintained in a temperature-controlled and a time-controlled lighting system. The handling and sacrifice of all animals were carried out in accordance with nationally prescribed guidelines, and ethical approval for the study was granted by the Animal Care and Use Committee (Kyushu University, Fukuoka, Japan).

### Immunohistochemistry of Mouse Tissues

All animals were deeply anesthetized with pentobarbital (50 mg/kg i.p.) and were perfused intracardially with saline followed by cold 4% paraformaldehyde in phosphate-buffered saline (PBS). Lumbar spinal cords were removed, immersed for at least12 h in the same 4% paraformaldehyde fixative at 4°C, and cryoprotected in 20 and 30% sucrose in PBS for at least12 h at 4°C. The tissues were embedded in OCT compound (Sakura Finetech, Tokyo, Japan), frozen and stored at -80°C until use. Cross sections (20 μm thickness) were cut by a cryostat, and collected as free-floating sections in PBS. Endogenous peroxidase was blocked by 3% H_2_O_2_ in methanol/PBS (1:1) for 30 min at room temperature (RT). After blocking with Block Ace (Dainippon Pharmaceutical, Osaka, Japan) for 30 min at RT, sections were incubated with diluted primary antibody at 4°C overnight. Then, they were processed using a Vectastain ABC kit (Vector Laboratories, Burlingame, CA, United States) with the appropriate biotinylated secondary antibody. The peroxidase reaction was detected using 3′3′-diaminobenzidine-tetrahydrochloride (Vector Laboratories).

### Indirect Immunofluorescence and Laser Scanning Confocal Microscopy

Cross sections (30 μm thickness) of mouse lumbar spinal cords were cut by a cryostat and collected as free-floating sections in PBS. They were incubated with a combination of primary antibodies at 4°C overnight. After rinsing, sections were incubated with the appropriate Alexa-Fluor-labeled secondary antibodies, and then counterstained with DAPI. Confocal images were acquired using a laser scanning confocal microscopy system (Nikon A1, Nikon, Tokyo, Japan). We used the sequential multiple fluorescence scanning mode to avoid non-specific overlap of colors, and captured all pictures under the same conditions of magnification, laser intensity, gain and offset values, and pinhole setting.

### Antibodies

We used two different antibodies against Cx36 (anti-Cx36), which were obtained from Thermo Fisher Scientific (Waltham, MA, United States). A rabbit polyclonal anti-Cx36 antibody (36-4600) was used for immunohistochemistry of human autopsied specimens (1:80) and western blot analysis of mouse tissues (1:1500). A mouse monoclonal anti-Cx36 antibody (37-4600) was used for immunohistochemistry (1:1,000) and immunofluorescence staining (1:150) of mouse tissues. The other antibodies for immunofluorescence were: rabbit polyclonal antibody against microtubule-associated protein 2 (MAP2) (M3696, 1:300, Sigma-Aldrich, St. Louis, MO, United States), rat monoclonal antibody against SOD1 (MABN834, 1:250, Millipore, Burlington, MA, United States), rabbit polyclonal antibody against zonula occludens-1 (ZO-1) (61-7300, 1:100, Thermo Fisher Scientific), guinea pig polyclonal antibody against vesicular glutamate transporter 1 (VGLUT1) (AB5905, 1:500, Millipore), and chicken polyclonal antibody against glutamate decarboxylase 65 (GAD65) (ab139958, 1:500, Abcam, Cambridge, United Kingdom). VGLUT1 was used as a marker of excitatory axon terminals, GAD65 was used as a marker of inhibitory axon terminals and MAP2 was used as a marker of dendrites. Secondary antibodies for immunofluorescence were: Alexa Fluor 488-conjugated goat anti-rabbit IgG antibody, Alexa Fluor 647-conjugated goat anti-rabbit IgG antibody, Alexa Fluor 546-conjugated goat anti-mouse IgG antibody, Alexa Fluor 405-conjugatd goat anti-mouse IgG antibody, Alexa Fluor 546-conjugated goat anti-rat IgG antibody, Alexa Fluor 488-conjugated goat anti-guinea pig IgG antibody, and Alexa Fluor 546-conjugated goat anti-chicken IgY antibody (Thermo Fisher Scientific).

### Synapse Classification

Multiple immunofluorescent stainings with anti-Cx36, anti-VGLUT1, anti-GAD65, and anti-MAP2 antibodies were performed on 15 sections of lumbar spinal cords from three mice per group (five sections from each mouse). Confocal immunofluorescence images of lamina IX were captured using a ×20 objective lens at the appropriate magnification to detect individual labeled puncta. Images were analyzed using the “Analyze particles” command in ImageJ. To avoid false positives, the threshold levels eliminating puncta that exist away from dendrites or neurons were decided for each channel. The outlines of each puncta were automatically drawn and Cx36-positive puncta inside neuronal cell bodies were manually eliminated because we wanted to analyze Cx36-positive puncta constructing GJs. The outlines of each channel were superposed and puncta were divided into seven groups according to the presence or absence of overlap: “Cx36 single positive (Cx36-S-Pos),” “VGLUT1 single positive (VGLUT1-S-Pos),” “GAD65 single positive (GAD65-S-Pos),” “Cx36/VGLUT1 double positive (Cx36/VGLUT1-D-Pos),” “Cx36/CAD65 double positive (Cx36/GAD65-D-Pos),” “VGLUT1/GAD65 double positive (VGLUT1/GAD65-D-Pos),” and “Cx36/VGLUT1/GAD65 triple positive (Cx36/VGLUT1/GAD65-T-Pos).” Counting was performed blind to the genotype and age of the mice.

### Western Blotting

Mouse lumbar spinal cords were homogenized in 200 μl of lysis buffer containing 200 mM Tris–HCl (pH 6.8), 8% sodium dodecyl sulfate (SDS), 40% glycerol, and 400 mM β-mercaptoethanol using a BioMasher (Nippi, Tokyo, Japan). After centrifugation at 10,000 ×*g* for 10 min, the supernatants were collected. The protein concentrations in the supernatants were measured using a DC protein assay Kit (Bio-Rad, Tokyo, Japan). Equal amounts of protein (20 μg each) were separated by 12% SDS-polyacrylamide gel electrophoresis. After electrophoresis, proteins were transferred electrophoretically onto polyvinyl difluoride membranes. The membranes were incubated with Block Ace (Dainippon Pharmaceutical), and subsequently incubated with anti-Cx36 antibody at 4°C overnight or with anti-β-actin antibody (mouse monoclonal, A5441, 1:20,000, Sigma-Aldrich) for 1 h at RT. After washing in Tris-buffered saline containing 0.1% Tween-20 (TBS-T), the membranes were incubated with a horseradish peroxidase-conjugated secondary antibody for 1 h at RT. The membranes were washed and visualized by an enhanced chemiluminescence system (ECL Prime, GE Healthcare Bio-Sciences AB, Uppsala, Sweden). Band intensities were measured using a ChemiDoc^TM^ XRS system (Bio-Rad Laboratories, Hercules, CA, United States) and normalized to β-actin levels.

### Real-Time Reverse Transcription (RT)-PCR Analysis

Total RNA was prepared from mouse lumbar spinal cords using ISOGEN (Nippongene, Tokyo, Japan). First-strand cDNA, which was synthesized using a ReverTra Ace qPCR RT Master Mix with gDNA Remover (Toyobo, Osaka, Japan) according to the manufacturer’s instructions, was subjected to quantitative PCR (qPCR). All qPCR assays were performed with a Thunderbird^TM^ SYBR^®^ Green qPCR MIX (Toyobo) and an Applied Biosystems 7500 Real-Time PCR System (Applied Biosystems, Thermo Fisher Scientific). The primers used to quantify the *Cx36* cDNA were 5′-TGATTGGGAGGATCCTGTTGAC-3′ and 5′-CATGGTCTGCTCATCATCGTAC-3′. The primers for *GAPDH* were 5′-AAATGGTGAAGGTCGGTGTG-3′ and 5′-TGAAGGGGTCGTTGATGG-3′. The expression levels of *Cx36* mRNA were determined by normalization to *GAPDH* mRNA, and the levels of *Cx36* mRNA relative to the wild type mice at each week of age are presented.

### Tissue Preparation and Immunohistochemistry of Autopsy Specimens

The Kyushu University Institutional Review Board for Clinical Research approved the study. Immunohistochemical studies were performed on autopsied lumbar spinal cord specimens from five ALS cases without family history and two non-neurological cases as a control. The clinical characteristics of the patients we examined in this study are summarized in Table 1. Three cases (ALS-1, 2, and 3) had bulbar onset, and two cases (ALS-4 and 5) had upper limb onset. The disease duration was relatively short in four cases (6 months to 2.5 years, ALS-1, 2, 3, and 4) and long in one case (7 years, ALS-5). The ALS-1 case with the shortest disease duration was still ambulatory at the time of death. Autopsy specimens were fixed in 10% buffered formalin and processed into paraffin sections. The sections were routinely treated with hematoxylin and eosin (H&E) stains. Five-micrometer-thick transverse sections were deparaffinized in xylene and dehydrated through an ethanol gradient. Endogenous peroxidase was blocked with 3% H_2_O_2_ in methanol/PBS (1:1) for 30 min at RT. After blocking with Block Ace (Dainippon Pharmaceutical) for 30 min at RT, sections were incubated with diluted primary antibody at 4°C overnight. Then, they were processed using a Vectastain ABC kit (Vector Laboratories) with the appropriate biotinylated secondary antibody. The peroxidase reaction was detected using 3′3′-diaminobenzidine-tetrahydrochloride (Vector Laboratories) and sections were counterstained with hematoxylin.

**Table 1:** Clinical characteristics of autopsied cases.

Case	Dx^a^	Age/Sex	Onset	Gait on death	Disease duration
Control-1	MI^b^	60/M	–	–	–
Control-2	Aspiration	80/M	–	–	–
ALS-1	ALS	75/M	Bulbar	Possible	6 months
ALS-2	ALS	60/M	Bulbar	Uncertain	11 months
ALS-3	ALS	75/M	Bulbar	Impossible	1.25 years
ALS-4	ALS	61/M	Upper limb	Impossible	2.5 years
ALS-5	ALS	64/F	Upper limb	Uncertain	7 years

*^*a*^Diagnosis. ^*b*^Myocardial infarction*.

### Statistical Analysis

Data are expressed as the mean ± SD. Pairwise comparisons between two groups were performed using Student’s *t*-test. *p* < 0.05 was considered statistically significant. All statistical analyses were carried out using JMP 9.02 software (SAS Institute, Cary, NC, United States).

## Results

### Cx36 Exists Independently of Chemical Synaptic Markers or Coexists With Chemical Synaptic Markers in the Lumbar Spinal Cord of Wild Type Mice

**FIGURE 1:**
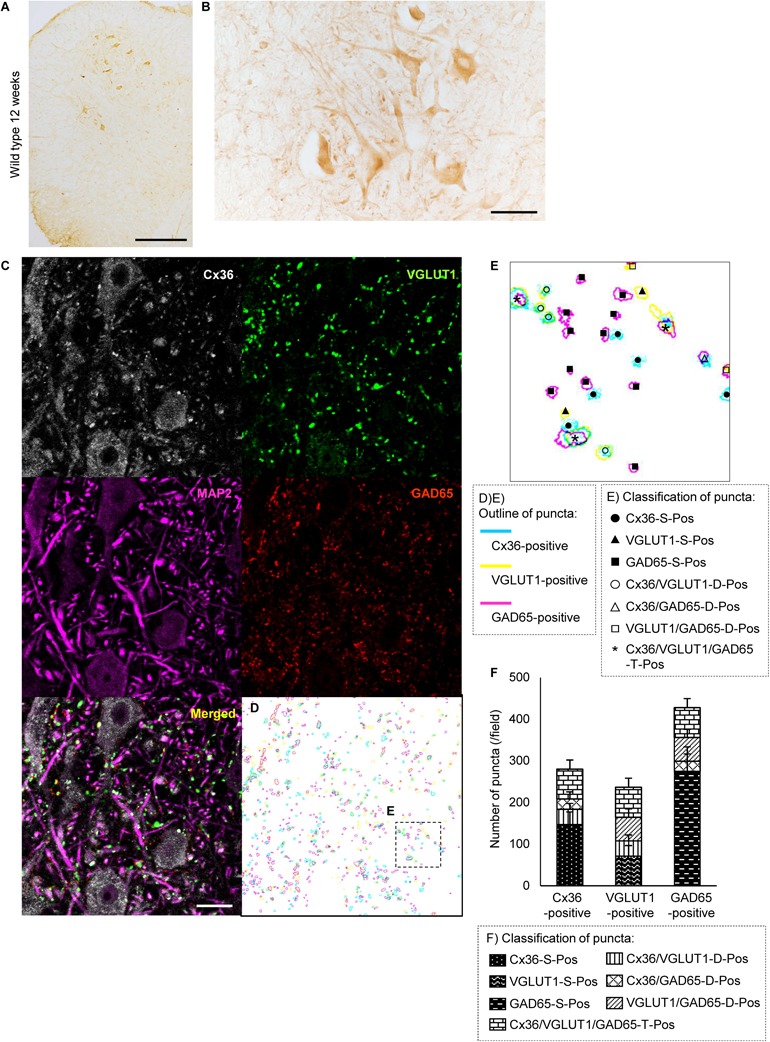
Distribution of Cx36 and synapse classification in the lumbar spinal cord of wild type mice at 12 weeks of age. **(A,B)** Immunohistochemical detection of Cx36 in the lumbar spinal cord of 12-week-old wild type mice. Cx36 IR was visible in both the anterior and posterior horns **(A)**. In the anterior horn, Cx36 IR was detected in neuronal somata, proximal dendrites, and neuropils **(B)**. Scale bars: 300 μm **(A)** and 50 μm **(B)**. **(C)** Confocal images of multiple immunofluorescent stainings. Cx36 (pseudocolored white), VGLUT1 (green), and GAD65 (red) IR were visible as puncta around neurons and along dendrites labeled with MAP2 (pseudocolored magenta). Scale bars: 20 μm. **(D)** Outline of each puncta. Pale blue line indicates Cx36-positive puncta, yellow line indicates VGLUT1-positive puncta, and magenta line indicates GAD65-positive puncta. **(E)** Higher magnification of the outlines [dotted-line box shown in **(C)**]. Puncta were classified into seven groups depending on the presence of overlap with other colored puncta. Explanation of the marks is described in the dotted-line box under the figure. **(F)** Fraction of the classified puncta. Explanation of the bar patterns is described in the dotted-line box under the graph. *N* = 15 fields from three mice. One field = 15,625 μm^2^. Error bars show the mean ± SD.

We first studied the distribution of Cx36 in the lumbar spinal cord of wild type mice by immunohistochemistry using anti-Cx36 antibody. Cx36 immunoreactivity (IR) was widely detected in both the anterior and posterior horns of the lumbar spinal cord (Figure 1A). In a high-power field of the anterior horn, we observed Cx36 IR in neuronal somata, proximal dendrites, and neuropils (Figure 1B).

To investigate the association between Cx36 and chemical synaptic markers in the lamina IX of the lumbar spinal cord, we performed multiple immunofluorescent stainings using anti-Cx36, anti-VGLUT1, anti-GAD65, and anti-MAP2 antibodies. Laser scanning confocal immunofluorescence microscopy detected Cx36 IR as puncta around neuronal somata and along MAP2-immunopositive dendrites (Figure 1C). Like Cx36 IR, VGLUT1, and GAD65 IR were detected as puncta around neuronal somata and at MAP2-immunopositive dendrites (Figure 1C). The colored outlines of each puncta obtained using the “Analyze particle” command in ImageJ (Figures 1D,E) were counted. We regarded puncta on the neuronal cell surface and along MAP2-immunopositive dendrites as significant signals that can construct channels or synapses on cell surfaces. We then classified these puncta into seven groups based on immunopositivity for Cx36, VGLUT1, and GAD65 (Figure 1E). In the lamina IX of wild type mouse lumbar spinal cord, 52.5 ± 5.8% of Cx36-positive puncta were Cx36-S-Pos, and they were assumed to be purely electrical synapses (Figure 1F). Other Cx36-positive puncta were assumed to exist as mixed synapses with chemical synapses labeled by VGLUT1 and/or GAD65. Of the Cx36-positive puncta, 13.3 ± 5.2% were Cx36/VGLUT1-D-Pos, 8.3 ± 4.9% were Cx36/GAD65-D-Pos, and 25.9 ± 7.5% were Cx36/VGLUT1/GAD65-T-Pos (Figure 1F). Of the VGLUT1-positive puncta, 45.9 ± 5.8 % existed with Cx36-positive puncta as Cx36/VGLUT1-D-Pos or Cx36/VGLUT1/GAD65-T-Pos (Figure 1F). GAD65-positive puncta were more likely to exist as GAD65-S-Pos and 22.5 ± 2.5% of GAD65-positive puncta existed with Cx36-positive puncta as Cx36/GAD65-D-Pos or Cx36/VGLUT1/GAD65-T-Pos (Figure 1F).

### Cx36 Expression Is Decreased in the Anterior Horn of the Lumbar Spinal Cord of 12-Week-Old SOD1^G93A^ Mice

**FIGURE 2:**
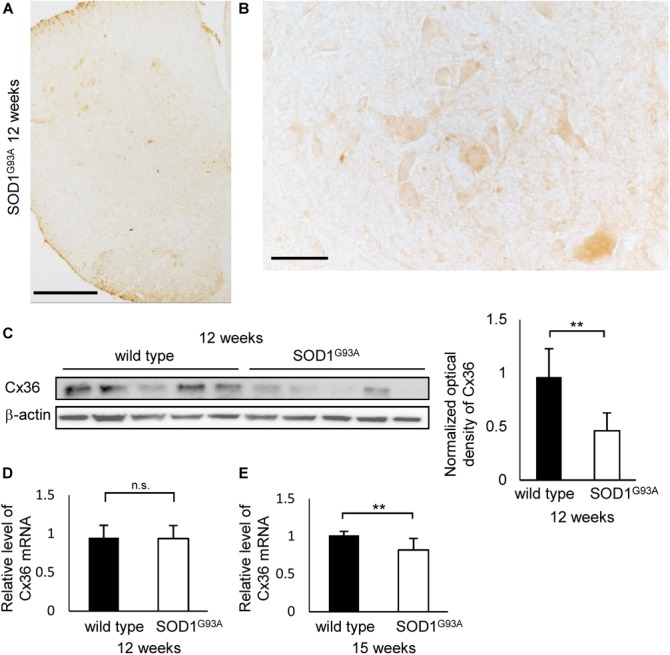
Expression of Cx36 in the lumbar spinal cord of SOD1^G93A^ mice at 12 weeks of age. **(A,B)** Immunohistochemical detection of Cx36 in the lumbar spinal cord of 12-week-old SOD1^G93A^ mice. Cx36 IR was diminished in the anterior horn and preserved in the posterior horn **(A)**. In a high-power field of the anterior horn, Cx36 IR was diminished in neuronal somata, dendrites and neuropils, although large motor neurons were still observed **(B)**. Scale bars: 300 μm **(A)** and 50 μm **(B)**. **(C)** Western blot analysis of lumbar spinal cord proteins from 12-week-old mice. The level of Cx36 protein in SOD1^G93A^ mice was significantly lower than that in wild type mice. *N* = 5 in each group. ^∗∗^*p* < 0.01 by Student’s *t*-test. Error bars show the mean ± SD. **(D,E)** RT-qPCR using mRNA from lumbar spinal cords. The relative levels of *Cx36* mRNA were not different between wild type and SOD1^G93A^ mice at 12 weeks of age **(D)**. At 15 weeks of age, the relative level of *Cx36* mRNA in SOD1^G93A^ mice was significantly lower than that in wild type mice **(E)**. *N* = 3 at 12 weeks of age, *N* = 5 at 15 weeks of age. ^∗∗^*p* < 0.01 by Student’s *t*-test. Error bars show the mean ± SD.

We examined the expression of Cx36 in the lumbar spinal cord of SOD1^G93A^ mice at 12 weeks of age, which were near the disease onset. Immunohistochemistry showed that Cx36 IR was decreased in neuronal somata, dendrites, and neuropils in the anterior horn of 12-week-old SOD1^G93A^ mice compared with 12-week-old wild type mice, although large motor neurons were still observed in the anterior horns of 12-week-old SOD1^G93A^ mice (Figures 1A,B, 2A,B). There was no significant difference in the posterior horns between wild type and SOD1^G93A^ mice at 12 weeks of age (Figures 1A,B, 2A,B). Quantitative western blot analysis using protein from lumbar spinal cords showed that the amount of Cx36 protein in 12-week-old SOD1^G93A^ mice was significantly lower than that in wild type mice at 12 weeks of age (Figure 2C). However, RT-qPCR using mRNA from lumbar spinal cords revealed that the relative level of *Cx36* mRNA was not different between wild type and SOD1^G93A^ mice at 12 weeks of age (Figure 2D). At 15 weeks of age, the relative level of *Cx36* mRNA in SOD1^G93A^ mice was significantly lower than that in wild type mice (Figure 2E).

### The Number of Cx36-Positive and GAD65-Positive Puncta Is Diminished in the Lamina IX of Lumbar Spinal Cord of 12-Week-Old SOD1^G93A^ Mice

**FIGURE 3:**
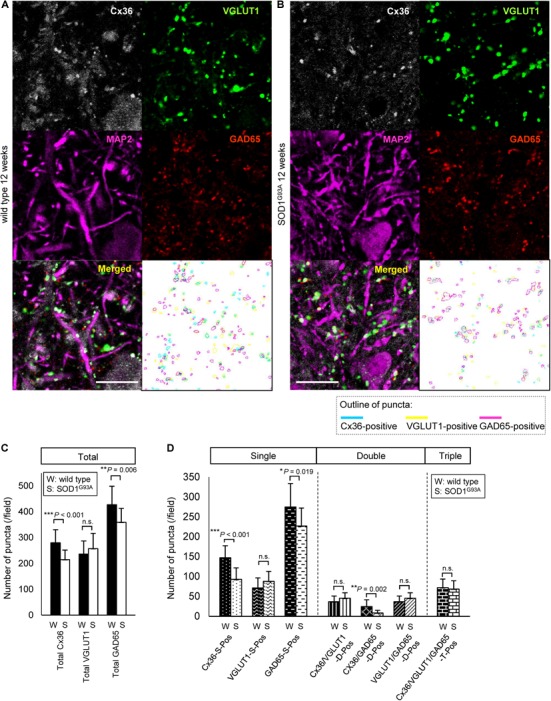
Comparison of the number of puncta immunopositive for Cx36 and/or chemical synaptic markers between wild type and SOD1^G93A^ mice at 12 weeks of age. **(A,B)** Confocal image and colored outline of each puncta in the lamina IX of the lumbar spinal cord of 12-week-old mice. Higher magnification of Figure 1C **(A)**. Cx36 (pseudocolored white), VGLUT1 (green), and GAD65 (red) IR were detected as puncta, and MAP2 (pseudocolored magenta) signals were preserved in SOD1^G93A^ mice **(B)**. Scale bar: 20 μm. **(C,D)** Comparison of the number of classified puncta between wild type and SOD1^G93A^ mice at 12 weeks of age. The total number of Cx36-positive puncta and GAD65-positive puncta was significantly decreased in SOD1^G93A^ mice **(C)**. As for colored outline overlap, the number of Cx36-S-Pos, GAD65-S-P, and Cx36/GAD65-D-Pos was significantly decreased in SOD1^G93A^ mice **(D)**. *N* = 15 fields from three mice per group. One field = 15,625 μm^2^. The *p* values by Student’s *t*-test are indicated in the graphs. Error bars show the mean ± SD.

Next, we compared the number of puncta on the neuronal cell surface and dendrites labeled by multiple immunofluorescent staining of lamina IX in the anterior horns. In 12-week-old SOD1^G93A^ mice, Cx36, VGLUT1, and GAD65 IR were detected as puncta and MAP2 IR was still observed (Figures 3A,B). The total number of Cx36-positive and GAD65-positive puncta was significantly decreased in SOD1^G93A^ mice, although there was no significant difference in the total number of VGLUT1-positive puncta (Figure 3C). As for colored outline overlap, the number of Cx36-S-Pos, GAD65-S-Pos, and Cx36/GAD65-D-Pos was significantly decreased in SOD1^G93A^ mice (Figure 3D). By contrast, there was no significant difference in the number of puncta that contained VGLUT1-positive puncta, such as VGLUT1-S-Pos, VGLUT1/Cx36-D-Pos, VGLUT1/GAD65-D-Pos, and Cx36/VGLUT1/GAD65-T-Pos between wild type and SOD1^G93A^ mice at 12 weeks of age (Figure 3D).

### Decrease in Cx36-Positive Puncta on Neuronal and Dendritic Surfaces Is Detected in SOD1^G93A^ Mice at 8 Weeks of Age

**FIGURE 4:**
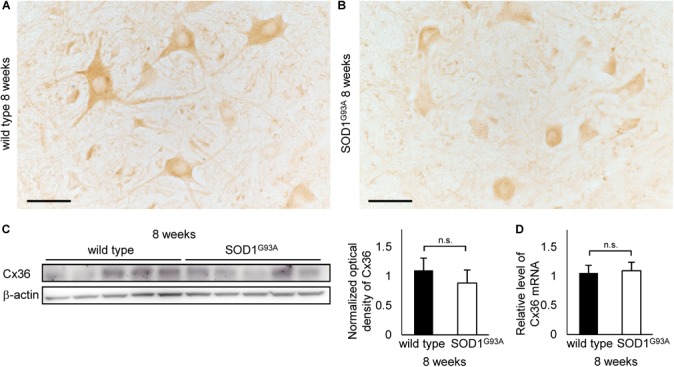
Cx36 expression in the lumbar spinal cord of 8-week-old mice. **(A,B)** Immunohistochemical detection of Cx36 in the anterior horn of wild type and SOD1^G93A^ mice at 8 weeks of age. Cx36 IR is seen in neuronal somata, proximal dendrites, and neuropils in wild type mice **(A)**, whereas Cx36 IR was diminished in proximal dendrites and neuropils in SOD1^G93A^ mice **(B)**. Scale bars: 50 μm. **(C)** Western blot analysis of lumbar spinal cord proteins from 8-week-old mice. The expression level of Cx36 protein in SOD1^G93A^ mice was lower than that in wild type mice, although the difference was not statistically significant (*p* = 0.154). *N* = 5 in each group. Error bars show the mean ± SD. **(D)** RT-qPCR using mRNA from lumbar spinal cords. The relative levels of *Cx36* mRNA were not different between wild type and SOD1^G93A^ mice at 8 weeks of age. *N* = 3. Error bars show the mean ± SD.

We examined 8-week-old mice to clarify whether the decrease in Cx36 expression is observed in SOD1^G93A^ mice at the presymptomatic stage. Using immunohistochemistry on 8-week-old wild type mice, Cx36 IR was detected in neuronal somata, dendrites, and neuropils, similarly to the 12-week-old wild type mice (Figure 4A). In 8-week-old SOD1^G93A^ mice, we observed a downward tendency in Cx36 IR in proximal dendrites and neuropils, but Cx36 IR in neuronal somata was preserved (Figure 4B). Quantitative western blot analysis using protein from mouse lumbar spinal cords showed that the amount of Cx36 protein was not significantly different between wild type and SOD1^G93A^ mice at 8 weeks of age (Figure 4C). In addition, RT-qPCR using mRNA from lumbar spinal cords revealed that the relative level of *Cx36* mRNA was not different between wild type and SOD1^G93A^ mice at 8 weeks of age (Figure 4D).

**FIGURE 5:**
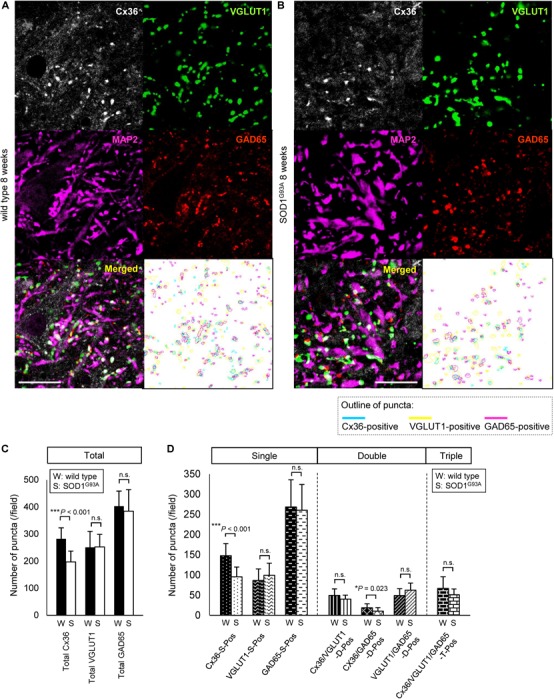
Comparison of the number of puncta immunopositive for Cx36 and/or chemical synaptic markers in the lumbar spinal cords between wild type and SOD1^G93A^ mice at 8 weeks of age. **(A,B)** Confocal images and colored outline of each puncta in the lamina IX of the lumbar spinal cord from 8-week-old mice. Cx36 (pseudocolored white), VGLUT1 (green), and GAD65 (red) IR were detected as puncta in both wild type mice **(A)** and SOD1^G93A^ mice **(B)**, and MAP2 (pseudocolored magenta) signals were preserved in SOD1^G93A^ mice **(B)**. Scale bar: 20 μm. **(C,D)** Comparison of the number of classified puncta between wild type and SOD1^G93A^ mice at 12 weeks of age. The total number of Cx36-positive puncta was significantly decreased in SOD1^G93A^ mice **(C)**. As for colored outline overlap, the number of Cx36-S-Pos and Cx36/GAD65-D-Pos was significantly decreased in SOD1^G93A^ mice **(D)**. *N* = 15 fields from three mice per group. One field = 15,625 μm^2^. The *p* values by Student’s *t*-test are indicated in the graphs. Error bars show the mean ± SD.

As it was difficult to detect subtle quantitative differences by western blot analysis using whole lumbar spinal cords, we performed multiple immunofluorescent stainings using lumbar spinal cords from 8-week-old wild type and SOD1^G93A^ mice, and compared the Cx36 puncta existing on neuronal and dendritic surfaces in lamina IX (Figures 5A,B). Compared with wild type mice, the total number of Cx36-positive puncta was significantly decreased in the lamina IX of lumbar spinal cords from 8-week-old SOD1^G93A^ mice, while there was no difference in the total number of VGLUT1-positive puncta and GAD65-positive puncta (Figure 5C). As for colored outline overlap, the number of Cx36-S-Pos and Cx36/GAD65-D-Pos was significantly decreased in 8-week-old SOD1^G93A^ mice, whereas the number of VGLUT1-S-Pos, GAD65-S-Pos, Cx36/VGLUT1-D-Pos, VGLUT1/GAD65-D-Pos, and Cx36/VGLUT1/GAD65-T-Pos was similar between wild type and SOD1^G93A^ mice (Figure 5D).

### ZO-1 Accumulation Is Detected in Motor Neurons Whose Expression of Cx36 Is Diminished

**FIGURE 6:**
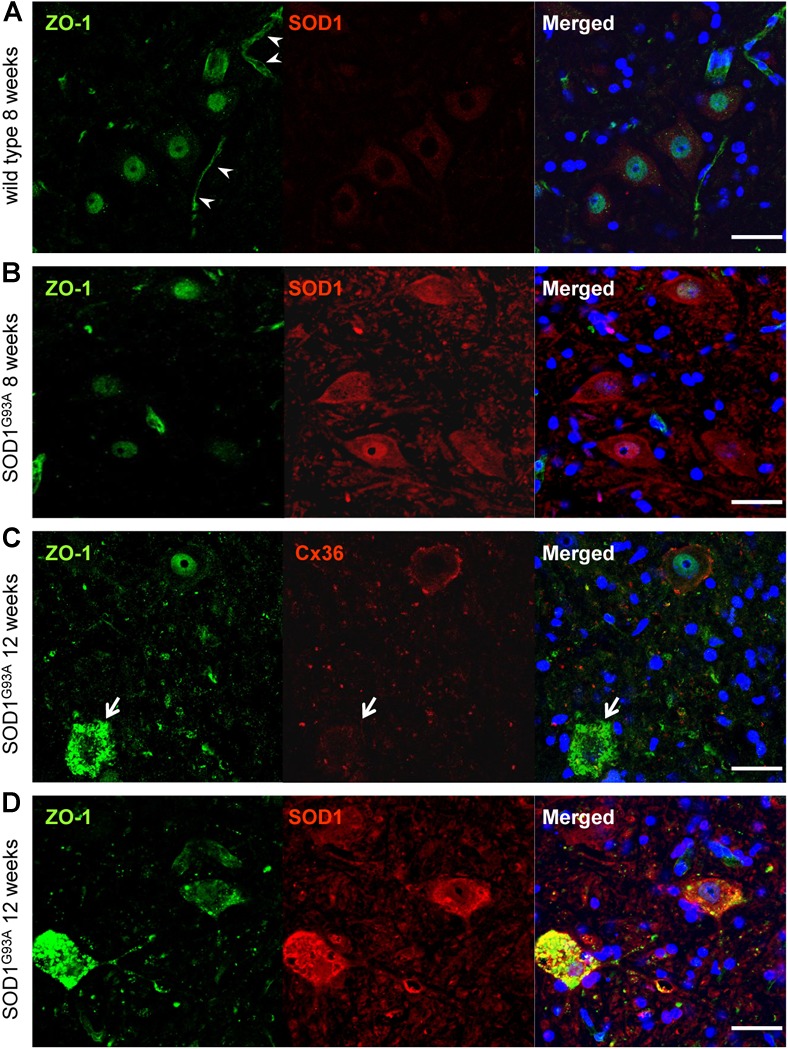
Cytoplasmic accumulation of ZO-1 in motor neurons of SOD1^G93A^ mice. **(A,B)** ZO-1 (green) IR was predominantly detected in the nucleus and was slightly immunopositive in the cytoplasm of large motor neurons in both wild type **(A)** and SOD1^G93A^ mice **(B)** at 8 weeks of age. In wild type mice, ZO-1 IR was also observed in vessel walls (arrowhead) **(A)**. **(C)** In 12-week-old SOD1^G93A^ mice, ZO-1 (green) was diffusely accumulated in the cytoplasm of spinal motor neurons, in which the expression of Cx36 (red) was diminished (arrow). **(D)** In 12-week-old SOD1^G93A^ mice, SOD1 (red) aggregation and ZO-1 (green) accumulation were partially colocalized in the cytoplasm of affected motor neurons at the anterior horns. Cell nuclei are counterstained with DAPI (blue). Scale bars: 50 μm.

Next, we examined ZO-1, which acts as a scaffolding protein for Cx36 in addition to being a tight junction protein constructing the blood–brain barrier (Li et al., 2004). Besides vessel walls, ZO-1 IR was predominantly observed in the nucleus and was slightly positive in the cytoplasm of large motor neurons in both wild type and SOD1^G93A^ mice at 8 weeks of age (Figures 6A,B). In 12-week-old SOD1^G93A^ mice, ZO-1 was diffusely accumulated in the cytoplasm of motor neurons whose expression of Cx36 was diminished (Figure 6C). Furthermore, in these mice, accumulated ZO-1 was partially colocalized with SOD1 aggregations that were observed in the cytoplasm of motor neurons (Figure 6D).

### Cx36 Immunoreactivity on Proximal Dendrites and Neuropils Is Decreased in Lumbar Spinal Cords From Patients With ALS

**FIGURE 7:**
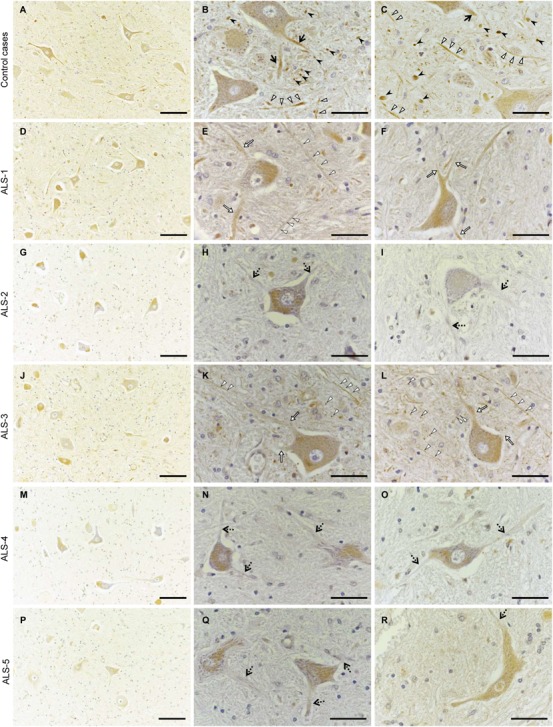
Decreased Cx36 IR in the anterior horn of patients with ALS. **(A–C)** Control cases. Cx36 IR was detected in proximal dendrites (black arrows) and neuropils as punctate (black arrowheads) or cord-like (white arrowheads) structures in the anterior horn from the myocardial infarction case **(A,B)** and the aspiration case **(C)**. **(D–R)** ALS cases. In ALS-1 and 3, a mild decrease in Cx36 IR was observed in both proximal dendrites (white arrows) and neuropils (dotted arrowheads) **(D–F,J–L)**. In ALS-2, 4, and 5, Cx36 IR was markedly diminished in both proximal dendrites (dotted arrows) and neuropils **(G–I,M–O,P–R)**. Scale bars: 100 μm **(A,D,G,J,M,P)** and 30 μm **(B,C,E,F,H,I,K,L,N,O,Q,R)**.

Finally, we performed immunohistochemistry on human autopsy tissues. In the anterior horns of the lumbar spinal cords from a myocardial infarction case and an aspiration case (control cases), Cx36 IR was detected in proximal dendrites and in neuropils as punctate or cord-like structures (Figures 7A–C). Cx36 IR was not observed in glial cells including astrocytes, oligodendrocytes, and microglia. We observed numerous large motor neurons in the anterior horns of the lumbar spinal cords from the four ALS cases with short disease durations (ALS-1, 2, 3, and 4; Figures 7D–O), while some large motor neurons were found in the anterior horns from ALS-5 with the longest disease duration (Figures 7P–R). In three cases (ALS-2, 4, and 5), Cx36 IR was markedly diminished in both proximal dendrites and neuropils (Figure 7G–I,M–R), and in two cases (ALS-1 and 3), a mild decrease in Cx36 IR was observed in both proximal dendrites and neuropils (Figure 7D–F,J–L).

## Discussion

The main new findings of the present study are: (1) in the anterior horns (lamina IX) of the lumbar spinal cord from wild type mice, about half of the Cx36 puncta existed independently of chemical synapse markers, while the rest coexisted with chemical synapse markers such as GAD65 and VGLUT1. (2) Neuronal and dendritic Cx36 puncta in the spinal anterior horns were significantly decreased in the early stage of SOD1^G93A^ ALS mice. (3) Cx36 single or Cx36/GAD65 double positive puncta, but not VGLUT1-containing puncta, were preferentially decreased in the anterior horns even at the pre-symptomatic stage of SOD1^G93A^ ALS mice. (4) In human sporadic ALS, neuronal and dendritic Cx36 IR was diminished in the relatively preserved large motor neurons in the lumbar anterior horns. These findings suggest that downregulation of neuronal and dendritic Cx36 in the spinal anterior horns occurs from the early stage of hereditary and sporadic ALS. The significant decrease in Cx36 protein levels in the lumbar cord of SOD1^G93A^ mice at 12 weeks of age (onset stage) in our study was consistent with a previous study using late stage SOD1^G93A^ ALS mice (Belousov et al., 2018). Although western blotting showed that the decrease in Cx36 protein levels was not statistically significant at 8 weeks of age (presymptomatic stage), we think it was because the whole spinal cord containing the posterior horns without any decrease in Cx36 IR was used. However, as the *Cx36* mRNA level was preserved in SOD1^G93A^ mice at both 8 and 12 weeks of age, the Cx36 decrease may be caused by translational suppression or increased degradation. It has been shown that the expression of Cx36 did not decrease upon overexpression of wild type SOD1 in cultured neurons from mouse spinal cord (Belousov et al., 2018), hence our findings in SOD1^G93A^ mice may be attributed to the G93A mutation rather than to the overexpression of SOD1 protein.

In the CNS, Cx36-made GJs exist independently or coexist with chemical synapses (Pereda, 2014). It has been reported that in the lamina IX of the anterior horn of the mouse lumbar spinal cord, 38% of Cx36-positive puncta morphologically form mixed synapses with primary afferent terminals, which are labeled with VGLUT1 and contacted by GAD65-positive terminals (Bautista et al., 2014). Our observation that 38.7% of Cx36-positive puncta colocalized with VGLUT1-positive puncta in wild type mice is consistent with these studies. Although Bautista et al. defined Cx36-positive puncta that were not colocalized with VGLUT1 as purely electrical synapses (Bautista et al., 2014), we detected Cx36/GAD65 double positive puncta in 8.3% of Cx36-positive puncta. Therefore, we consider Cx36 single positive puncta that exist with neither VGLUT1 nor GAD65 as purely electrical synapses.

Electron microscopy demonstrated that axodendritic synapses are affected earlier than motor neuron soma and axons in the lumbar spinal cord of SOD1^G93A^ mice (Vinsant et al., 2013). Synaptophysin-immunoreactive presynaptic boutons on somata and proximal dendrites of motor neurons are significantly decreased at P90, but not at P60, in the lumbar spinal cord of SOD1^G93A^ mice with a B6/SJL background (Zang et al., 2005), which exhibit a faster disease progression than SOD1^G93A^ mice with a C57BL/6 background used in the present study (Heiman-Patterson et al., 2005). Accordingly, it is possible that Cx36-made electrical synapses are impaired earlier than or as early as chemical synapses in the anterior horns of the lumbar spinal cord of SOD1^G93A^ mice.

The decrease in Cx36 single positive puncta and Cx36/GAD65 double positive puncta without any decrease in Cx36/VGLUT1 double positive and Cx36/VGLUT1/GAD65 triple positive puncta suggests that purely electrical synapses and mixed synapses without glutamatergic axon terminals are more vulnerable than mixed synapses containing glutamatergic axon terminals in SOD1^G93A^ mice. Although the downregulation mechanism of Cx36 single positive puncta and Cx36/GAD65 double positive puncta remains unknown, it has been shown that coexisting glutamatergic synapses potentiate electrical transmission of Cx36-made synapses *via* activation of *N*-methyl-D-aspartate receptors or metabotropic glutamate receptors (Wang et al., 2010; Pereda, 2014). Therefore, the coexistence of glutamatergic synapses may render Cx36/VGLUT1 double positive puncta and Cx36/VGLUT1/GAD65 triple positive puncta more resistant, whereas the absence of such glutamatergic inputs may render Cx36-made electrical synapses and Cx36/GABAergic mixed synapses more vulnerable. It has been reported that Cx36 is essential for synchronous activity of inhibitory networks in the mouse cortex (Deans et al., 2001) and that motor neurons control electrical activity of neighboring neurons *via* Cx36-made electrical synapses (Song et al., 2016). In the mouse spinal cord, Cx36 is associated with presynaptic inhibition of primary afferents because Cx36-deficient mice exhibit loss of sensory-evoked presynaptic inhibition (Bautista et al., 2012). The expression of Cx36 in the rat spinal cord was decreased 7 days after spinal cord injury, and therapeutic administration of modafinil, which increases electrical coupling, normalized the neurological symptoms including hyper-reflexia and spasticity at the chronic phase (Yates et al., 2011). These reports indicate that Cx36 plays a key role in interneuronal communication, especially in inhibitory systems. As the effect of an early decrease in Cx36 single positive puncta and Cx36/GAD65 double positive puncta on synaptic activity in SOD1^G93A^ mice was not examined, further research is required to conclude that the loss of Cx36-made electrical synapses is involved in neurodegeneration in ALS. If impairment of Cx36-made electrical synapses causes dysregulation between neurons and induces hyperexcitability of motor neurons, subsequent motor neuronal death and altered output to innervated muscles, which is detected by needle electromyography in ALS, may occur. Thus, restoration of electrical synapses in motor neurons may be a potential therapeutic target for motor neuron diseases. Alternatively, downregulation of Cx36 puncta may be a host defense mechanism, because it has recently been demonstrated that knockdown of Cx36 in cultured motor neurons from wild type mice reduced neuronal death caused by overexpression of the mutant human SOD1-G93A protein (Belousov et al., 2018).

In the present study, we also detected cytoplasmic accumulation of ZO-1, a scaffolding protein for Cx36 (Li et al., 2004), in motor neurons of SOD1^G93A^ mice at 12 weeks of age. However, the absence of ZO-1 accumulation in motor neurons of SOD1^G93A^ mice at 8 weeks of age suggests that the decrease in neuronal Cx36 precedes the accumulation of ZO-1, thus the aggregation of ZO-1 does not induce Cx36 puncta loss. Instead, downregulation of Cx36 may contribute to the formation of the secondary cytoplasmic accumulation of ZO-1 protein, leading to motor neuron dysfunction in SOD1^G93A^ mice.

The physiological and pathological roles of Cx36-made electrical synapses in the human spinal cord are not totally unknown. The decrement of Cx36 IR in the lumbar spinal cord of all ALS cases examined in our study is consistent with previous western blot results showing a decrease in Cx36 protein levels in the ALS spinal cord (Belousov et al., 2018). The previous study did not confirm the existence of motor neurons in the spinal cord, whereas we identified the presence of many large morphologically well-preserved anterior horn cells in ALS patients with bulbar or upper limb onset, who had lower limb involvement later. Although we have not identified a relationship between Cx36 positive puncta and chemical synaptic markers in the human spinal cord, yet, Cx36-made electrical or mixed synapses may be involved in human sporadic ALS, especially in the early degenerative stage of motor neuron cells.

Our study has several limitations. First, false classification of synapses is possible because we analyzed synaptic characteristics using three antibodies as synaptic markers and laser scanning confocal microscopy. If each marked punctum of an identical mixed synapse exists at different focal planes of the confocal microscope, the mixed synapse may appear as a single or double positive punctum. Axon terminals may be labeled by synaptic markers other than those used in the present study, which may form a mixed synapse with Cx36-positive puncta, although VGLUT2-positive axonal terminals, which are abundant around motor neurons (Bautista et al., 2014), did not colocalize with Cx36-positive puncta. Second, Cx36 hemichannels are possibly present in Cx36-positive puncta. Although it is still controversial that Cx36 hemichannels play either a pro-death or pro-survival role in neuronal death (Schock et al., 2008; Fontes et al., 2015; Orellana et al., 2011; Belousov et al., 2017), Cx36 hemichannels are expected to exist in the process of Cx36 downregulation and be involved in neuronal degeneration. Finally, we did not perform quantitative analysis of Cx36-positive puncta in human ALS samples. Thus, our Cx36 IR findings in human ALS should be regarded as preliminary. Further studies are required to confirm that Cx36-made electrical synapses are impaired in human ALS, and play roles in motor neuron degeneration.

In summary, we reported the decrease in neuronal and dendritic Cx36 puncta in the early stage of the SOD1^G93A^ mouse ALS model. Interestingly, morphologically well-preserved large motor neurons in the spinal anterior horns from sporadic ALS patients also showed decreased Cx36 IR. The SOD1^G93A^ mouse is a model of familial ALS with the *SOD1* mutation whose pathogenic mechanism is not identical to that of human sporadic ALS. Our findings suggest that the loss of Cx36-made electrical synapses may be a common process of neuronal degeneration in both familial and sporadic ALS. This decline is the opposite response to the upregulation of Cx36-made electrical synapses that has been reported in acute neuronal injury (Oguro et al., 2001; Gajda et al., 2003; de Pina-Benabou et al., 2005; Garrett and Durham, 2008; Wang et al., 2012). A specific pathomechanism inducing the downregulation of Cx36 expression may exist in motor neuron diseases. Further functional investigation of electrical synapses may lead to novel therapeutic strategies for ALS.

## Author Contributions

YK, KM, RY, and J-iK designed the study and drafted the manuscript. YK, KM, WS, and Sho-H performed the experiments. Shi-H and KO provided key samples and scientific suggestions. YK, KM, RY, TM, and J-iK analyzed the data. All authors reviewed the manuscript.

## Conflict of Interest Statement

RY has received honoraria from Biogen Japan and Japan Blood Products Organization. TM has received honoraria from Bayer Healthcare, Biogen Japan, Takeda Pharmaceutical Co., Ltd., and Mitsubishi Tanabe Pharma. J-iK is a consultant for Biogen Japan and Medical Review and has received honoraria from Bayer Healthcare, Mitsubishi Tanabe Pharma, Nobelpharma, Otsuka Pharmaceutical, Sanofi K.K., Chugai Pharmaceutical Co., Ltd., Teijin Pharma, Novartis Pharma, and Medical Review. The remaining authors declare that the research was conducted in the absence of any commercial or financial relationships that could be construed as a potential conflict of interest.
